# Optimising the yield from bronchoalveolar lavage on human participants in infectious disease immunology research

**DOI:** 10.21203/rs.3.rs-2505850/v1

**Published:** 2023-02-02

**Authors:** Jane Alexandra Shaw, Maynard Meiring, Devon Allies, Lauren Cruywagen, Tarryn-Lee Fisher, Kesheera Kasavan, Kelly Roos, Stefan Marc Botha, Candice MacDonald, Andriëtte M. Hiemstra, Donald Simon, Ilana van Rensburg, Marika Flinn, Ayanda Shabangu, Helena Kuivaniemi, Gerard Tromp, Stephanus T. Malherbe, Gerhard Walzl, Nelita du Plessis, Stellenbosch University Immunology Research Group Bronchoalveolar Lavage Study Consortium (SU IRG BAL Study Consortium)

**Affiliations:** Biomedical Research Institute, Division of Molecular Biology and Human Genetics, Faculty of Medicine and Health Sciences, Stellenbosch University; Biomedical Research Institute, Division of Molecular Biology and Human Genetics, Faculty of Medicine and Health Sciences, Stellenbosch University; Biomedical Research Institute, Division of Molecular Biology and Human Genetics, Faculty of Medicine and Health Sciences, Stellenbosch University; Biomedical Research Institute, Division of Molecular Biology and Human Genetics, Faculty of Medicine and Health Sciences, Stellenbosch University; Biomedical Research Institute, Division of Molecular Biology and Human Genetics, Faculty of Medicine and Health Sciences, Stellenbosch University; Biomedical Research Institute, Division of Molecular Biology and Human Genetics, Faculty of Medicine and Health Sciences, Stellenbosch University; Biomedical Research Institute, Division of Molecular Biology and Human Genetics, Faculty of Medicine and Health Sciences, Stellenbosch University; Biomedical Research Institute, Division of Molecular Biology and Human Genetics, Faculty of Medicine and Health Sciences, Stellenbosch University; Biomedical Research Institute, Division of Molecular Biology and Human Genetics, Faculty of Medicine and Health Sciences, Stellenbosch University; Biomedical Research Institute, Division of Molecular Biology and Human Genetics, Faculty of Medicine and Health Sciences, Stellenbosch University; Biomedical Research Institute, Division of Molecular Biology and Human Genetics, Faculty of Medicine and Health Sciences, Stellenbosch University; Biomedical Research Institute, Division of Molecular Biology and Human Genetics, Faculty of Medicine and Health Sciences, Stellenbosch University; Biomedical Research Institute, Division of Molecular Biology and Human Genetics, Faculty of Medicine and Health Sciences, Stellenbosch University; Biomedical Research Institute, Division of Molecular Biology and Human Genetics, Faculty of Medicine and Health Sciences, Stellenbosch University; Biomedical Research Institute, Division of Molecular Biology and Human Genetics, Faculty of Medicine and Health Sciences, Stellenbosch University; Biomedical Research Institute, Division of Molecular Biology and Human Genetics, Faculty of Medicine and Health Sciences, Stellenbosch University; Biomedical Research Institute, Division of Molecular Biology and Human Genetics, Faculty of Medicine and Health Sciences, Stellenbosch University; Biomedical Research Institute, Division of Molecular Biology and Human Genetics, Faculty of Medicine and Health Sciences, Stellenbosch University; Biomedical Research Institute, Division of Molecular Biology and Human Genetics, Faculty of Medicine and Health Sciences, Stellenbosch University

**Keywords:** bronchoalveolar lavage, bronchoscopy, tuberculosis, research methods bronchoalveolar lavage, bronchoscopy, tuberculosis, research methods

## Abstract

Bronchoalveolar lavage (BAL) is becoming a common procedure for research into infectious disease immunology. Little is known about the clinical factors which influence the main outcomes of the procedure. In research participants who underwent BAL according to guidelines, the BAL volume yield, and cell yield, concentration, viability, pellet colour and differential count were analysed for association with important participant characteristics such as active tuberculosis (TB) disease, TB exposure, HIV infection and recent SARS-CoV-2 infection. In 337 participants, BAL volume and BAL cell count were correlated in those with active TB disease, and current smokers. The right middle lobe yielded the highest volume. BAL cell and volume yields were lower in older participants, who also had more neutrophils. Current smokers yielded lower volumes and higher numbers of all cell types, and usually had a black pellet. Active TB disease was associated with higher cell yields, and higher proportions of granulocytes, but this declined at the end of treatment. HIV infection was associated with lower cell yields and more bloody pellets, and recent SARS-CoV-2 infection with a higher proportion of lymphocytes. These results allow researchers to optimise their participant and end assay selection for projects involving lung immune cells.

## Introduction

**Figure 1:**
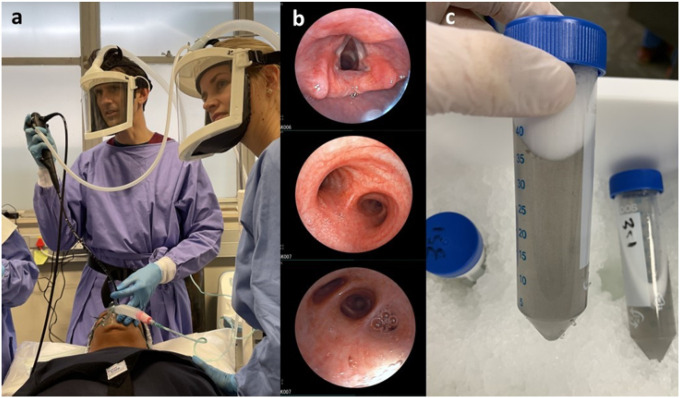
Bronchoscopy and bronchoalveolar lavage. (A) A research participant is about to have a Fujifilm flexible videoscope with 3.2 mm working channel inserted through her nose while under conscious sedation. Clinicians performing the procedure are authors STM and JAS. (B) Sequential photos from within the airways (from top to bottom: vocal cords before intubation, main carina, and right middle lobe medial segment subsegmental bronchi) taken with the bronchoscope. (C) Results of the bronchoalveolar lavage, aliquots of saline with foamy surfactant on the surface and in this case significant staining with black particulate matter from tobacco smoking.

Bronchoscopy with bronchoalveolar lavage (BAL) is a commonly performed clinical procedure that yields fluid containing cells and other substances that populate the lung mucosa and alveolar surface, assisting in diagnosis and treatment of many lung diseases. A flexible video bronchoscope introduced into a participant’s airway, advanced until gently wedged in a target lung lobe or segment, allows warmed sterile saline to be instilled and then aspirated through the working channel of the bronchoscope (Fig. 1).

The procedure is not without risk, as often the patient needs intravenous (IV) sedative agents, there may be mechanical trauma from the scope or endobronchial procedures which can cause bleeding or pneumothorax, or residual fluid which cannot be aspirated may, rarely, cause problems such as infection^1^. These risks can be mitigated and therefore BAL for research alone, without any envisioned clinical benefit to the participant, is becoming a common procedure. The fluid is used for both qualitative and quantitative measurements to study structural and immune cells, soluble mediators, mucus, and features of tissue remodelling, at the site of disease^2^. Researchers have an ethical duty to ensure that the procedure is as safe as possible for the volunteer, and that the BAL specimen retrieved is of sufficient quantity and quality to be of scientific value. The European Respiratory Society (ERS) in 1999 and the American Thoracic Society (ATS) in 2012 published guidance documents (the latter specifically for patients with suspected interstitial lung disease), in an attempt to standardise BAL procedures, processing and analysis^3,4^. Research studies may, however, require deviations from these recommendations, and many other factors can influence the outcomes of a BAL procedure beyond these parameters. For example, underlying chronic obstructive pulmonary disease (COPD) is associated with lower BAL volume yield than that from controls, and the yield is worse with declining lung function and disease severity on imaging^5^·^6^; tobacco and cannabis smoking both have been shown to increase BAL cell yields^7,8^; and a manual aspiration technique improves volume and cell yield over wall suction use^9^. Moreover, there are likely to be many factors which influence outcomes that are yet unknown to us. At the time of the bronchoscopy, the only measures of success or failure the physician (operator) has, is the wellbeing of their patient and the BAL volume yield, or percentage of instilled volume returned. It is only after processing of the specimen that the feasibility of using the BAL for specific research assays can be established, as determined by the cell quantity, quality, viability and differential count.

The aim of this study was to optimise bronchoscopy with BAL for immunology research, by identifying factors which affected the main BAL outcomes when procedures are performed according to current recommendations: BAL volume yield, total BAL cell yield and cell concentration, BAL cell viability, pellet colour (to identify blood contamination) and differential cell counts. In addition, procedure-related adverse events during the bronchoscopy were investigated. Special attention was given to investigating the effects (if any) of infections on BAL outcomes, specifically tuberculosis (TB), HIV infection and recent SARS-CoV-2 infection.

## Methods

### Study Design

Participants in this study were recruited from five separate National Institutes of Health (NIH)-funded projects in which BAL was performed between 1 January 2019 and 15 March 2022, for research into host immune responses to *Mycobacterium tuberculosis* (Mtb) by the Biomedical Research Institute (BMRI) Clinical Team and the Immunology Research Group (IRG) laboratory of Stellenbosch University, Cape Town, South Africa. These studies included participants recruited through contact with BMRI community-based research workers from subdistricts in the Cape Town metropolitan region, as well as from the Drakenstein and Saldanha municipal subdistricts of the Western Cape. Data on clinical parameters were collected by research clinicians and nurses as part of the studies in which the participants were enrolled, while the laboratory processing data were collected by the research assistants on the day of processing. All participants provided written informed consent for the parent study into which they were enrolled. Each parent study was approved by the Stellenbosch University Health Research Ethics Committee (reference numbers: N16/03/033, N18/10/118, N19/07/093, N19/10/150 and N20/07/079). Research was performed in accordance with the Declaration of Helsinki. The participant and authors in Fig. 1 have provided informed consent to publish the image in an online open access publication.

In general, participants were included if they met the following criteria: 1) age 18–65 y; 2) weight 35 kg-120 kg; 3) absence of current source of infection apart from those required for specific groups; 4) consented to bronchoscopy with BAL, Positron Emission Tomography (PET)-CT scan where applicable, and nasopharyngeal swab for SARS-CoV-2 reverse transcription quantitative real time-Polymerase Chain Reaction (RT-qPCR); and 5) had a verifiable address or residence location for duration of the study. Exclusion criteria included: 1) pregnancy or breastfeeding; 2) haemoglobin < 9.0 g/dl; 3) any severe systemic condition/co-morbidity that may affect the safety of the participant or the performance of the assays; 4) diabetes (point of care HbA1c ≥ 6.5%, random glucose ≥ 200 mg/dl (or 11.1 mmol/L), fasting plasma glucose ≥ 126 mg/dl (or 7.0 mmol/L), or the concomitant use of any anti-diabetic agent); 5) immunosuppressive medication use, including inhaled corticosteroids, within the past 2 weeks; 6) substance or alcohol abuse that in the opinion of the investigator may interfere with the participant’s adherence to study procedures; or any person that the physician/study nurse feels is not appropriate for the study, or that the study is not in the person’s best interest; 7) known allergy or intolerance to drugs used in procedural sedation or a known history or family history of malignant hyperthermia; or 8) history of taking antibiotics with anti-TB activity in the past 4 weeks (non-TB groups).

Participants were divided into the following groups according to history, clinical assessment, imaging and laboratory investigations:

1. TB groups: categorised according to treatment duration into ‘Pre-Treatment’(treatment not yet initiated), ‘Early Treatment’ (less than one month) and ‘End of Treatment’ (completed six months of standard treatment). Participants with drug resistant TB or extrapulmonary TB (without predominant intrathoracic TB component) were excluded.
2. Household Contacts: close contact with an individual with active untreated TB within three months; active TB disease ruled out on sputum testing, chest radiograph and symptom screen. Contacts were excluded if they had previous TB disease in the last three years, or current TB symptoms.
3. Community Controls: no active TB disease in previous three years, no recent known exposure to TB, active TB ruled out on sputum testing, chest radiograph and symptom screen.
4. People living with HIV (PLHIV): known or newly diagnosed HIV infected individuals, active TB disease ruled out on sputum testing, chest radiograph and symptom screen.

### Research procedures

#### Mtb detection

All participants had sputum testing for Mtb with Auramine smear, liquid culture [Mycobacterial Growth Indicator Tubes (MGIT), BACTEC 960, Becton Dickinson], and GeneXpert MTB/RIF Ultra^®^ assay (Cepheid, Sunnyvale, CA, USA), hereafter ‘Xpert Ultra’. A diagnosis of TB was assigned if a symptomatic individual had a positive MGIT culture or Xpert Ultra (graded as medium and higher), or positive Xpert Ultra graded as lower than medium but with consistent chest radiograph features.

All participants except those with active TB disease were also tested with an interferon gamma release assay (IGRA), in this case QuantiFERON-TB Gold Plus^®^ (QFT) assay (Qiagen, Carnegie, Australia).

#### SARS-CoV-2 detection

The SARS-CoV-2 positive subgroup was defined as a previous verifiable positive SARS-CoV-2 RT-qPCR in a local reference laboratory, or positive serology on the Abbott SARS-CoV-2 IgG assay (Abbott Laboratories, South Africa, Pty Ltd) or the Abbott SARS-CoV-2 IgG II Quant (Abbott Laboratories, South Africa, Pty Ltd), both done on the Architect i System (Abbott Laboratories, Illinois, USA). All SARS-CoV-2 tests were carried out by the National Health Laboratory Service (NHLS) of South Africa (https://www.nhls.ac.za/). The strategy interpreting the serology results is described in Supplementary Table S1, in accordance with study protocol^10^. Participants who were previously vaccinated against SARS-CoV-2 who only had a positive anti-S antibody on serologic testing, and no previous positive RT-qPCR (and therefore no evidence of infection-acquired immunity or previous infection), were not included in the SARS-CoV-2 positive subgroup.

#### Bronchoscopy with bronchoalveolar lavage (BAL)

Procedures were performed in accordance with current recommendations^34^. In brief, all participants were pre-screened for fitness for bronchoscopy according to predefined criteria by a study clinician with knowledge of the procedure. A dedicated sedation practitioner achieved conscious sedation using either a propofol/fentanyl or midazolam/fentanyl combination. The participant received pre-oxygenation and continuous supplemental oxygen throughout the procedure, with continuous cardiac, non-invasive blood pressure and peripheral saturation monitoring until full recovery. Topical 1% lignocaine spray was applied to the throat and an anaesthetic gel in the nose. Topical lignocaine was applied to the vocal cords and large airways through the working channel of the flexible video bronchoscope which was then gently wedged in the target segment. Sequential aliquots of 60 ml were individually instilled then manually aspirated with a 20 ml syringe after ten seconds ‘dwell time’, to a total of 240 ml. The target lobe was chosen by the team prior to the procedure, based on the protocol of the parent study. In general the right middle lobe (RML) was targeted in Community Controls and Household Contacts with normal chest imaging (with lingula as backup), and the area of most involvement with pulmonary TB or an imaging abnormality in keeping with subclinical TB in a Household Contact was targeted. Two of the studies included bronchial brushings (a single use brush with fine silicone bristles was used to harvest cells and microbiological material by lightly abrading the bronchial mucosa when inserted through the bronchoscope working channel) as part of their procedure, done either before or after the BAL depending on the standard operating procedure at the time. A thoracic ultrasound was performed in the recovery room on participants who underwent brushings, to exclude complications.

#### Adverse events

Data on adverse events were collected from an additional 20 study participants, resulting in 357 BAL procedures being included in these analyses. Information on adverse events was collected during the procedure and in recovery by clinicians using standardized patient record sheets. Participants were also contacted by study staff at 72 h and 14 days after the procedure to gather information regarding adverse events and persistent symptoms using a standardised list of questions, captured directly onto the electronic database. If needed a follow up review was arranged.

#### Processing of BAL fluid

The BAL fluid was placed on ice immediately after aspiration. Processing was initiated within two hours of harvest. Steps differed between studies, but in general the processing steps included: pooling of all aliquots, two sterile filtration steps (Falcon Cell Strainers 70 μm and 40 μm, Corning Inc., NY), then centrifugation for ten minutes at 350 × g at + 8°C to pellet the cells. Two washing steps were performed using supplemented Roswell Park Memorial Institute Medium (RPMI 1640; Biowest, Nuaill6, France), that is 46.5 ml RPMI 1640 with 500 μI of 1% L-glutamine (MilliporeSigma, Massachusetts, USA), 2.5 ml of 5% Human AB serum (MilliporeSigma, Massachusetts, USA), and 500 μ! of a 1% Penicillin/Streptomycin/Amphotericin B mixture [100 lU/ml Penicillin, 100 μg/ml Streptomycin and 0.25 μg/ml Amphotericin B in 0.85% saline (MilliporeSigma, Massachusetts, USA)]. If the pellet was judged contaminated with blood by visual inspection, an additional red cell lysis step was performed in 1 ml of Lonza ACK lysis buffer (1x) [Whitehead Scientific (Pty) Ltd, SA]. The total cell count was done with a haemocytometer and viability check by Trypan Blue exclusion method. A fraction (1 % of the total cell count) was placed in the cytocentrifuge for the Cytospin (Simport, Saint-Mathieu-de-Beloeil, Canada) and differential count procedure. The Cytospin slide (ELITechGroup, Paris, France) was fixed with a fixative and stained RAPI-DIFF Stain 1 (contains Eosin Y dye in phosphate buffer) and using RAPI-DIFF Stain 2 (Clinical Sciences Diagnostics, Johannesburg, South Africa) containing polychromed methylene blue in phosphate buffer for 30s each. Cytospin slides were counted manually (to a total of 100 cells). The BAL was finally fractionated according to the study protocol and cryopreserved in 10% dimethyl sulfoxide (MilliporeSigma, Massachusetts, USA) and 90% fetal bovine serum (Cytiva, Massachusetts, USA) and, by gradual cooling to −80°C in a Nalgene Mr Frosty^™^ container [Sigma Aldrich (Pty) Ltd, Gauteng, South Africa] with isopropanol for 24 h followed by long term preservation in liquid nitrogen.

#### PET-CT scans

Household Contacts, PLHIV, and a subset of the Community Controls had PET-CT imaging performed in addition to the routine chest radiographs, according to their study protocol. PET-CT imaging was done at the Nuclear Medicine Research Institute Node for Infection Imaging (NII), Stellenbosch University, South Africa with a Vereos PET-CT scanner (Philips, Amsterdam, Netherlands).^10^ 18F-fluorodeoxyglucose (FDG) radiotracer was administered at a dose of 2.8 mBq per kg, 60 min before imaging. Results were reported by consensus between a nuclear medicine physician and a radiologist.

#### Analyses

Analyses were conducted in R.^11^ Simple descriptive statistics were used to describe the observations, with percentages and proportions for categorical variables, and means with standard deviations or medians with interquartile ranges for continuous data that had a normal and skewed distribution respectively.

The outcomes of interest in the analysis were determined as:

1. BAL volume yield: reported in millilitres of fluid or as the percentage of instilled volume returned with aspiration. An optimal yield from BAL is >30% of the instilled volume (i.e. > 70 ml from a 240 ml BAL)^4^.
2. Total BAL cells (reported as counts) and BAL cell concentration (total cells per millilitre of BAL fluid), and viability (percentage of live cells in total cell count).
3. Pellet colour: categorised as bloody, clear, or black. Of particular importance is the bloody pellet, which implies contamination with blood and therefore trauma to the mucosal surface (a minor adverse event). A bloody pellet requires a cell lysis step during processing to lyse erythrocytes, as the presence of red blood cells may reduce the viability of the immune cells after thawing, and as the cells originate in the peripheral blood rather than the lung, they potentially undermine the results of the immunological end assays done on the BAL fluid.
4. Differential cell count in BAL fluid: reported as the relative proportions of macrophages (normal > 85%), lymphocytes (normal 10–15%), neutrophils (normal ≤ 3%) and eosinophils (normal ≤ 1%). The pattern of abnormality may provide insight into the underlying immune dysfunction^4^.

For the above outcomes, simple linear regression models were supplemented with a modified random forest model to identify variables with an appreciable effect on the outcome of interest from any procedure. For each variable, data were fed into the Boruta algorithm, an all-relevant feature selection algorithm to filter out those predictors which do not have a significant impact on the outcome^12^. The variables identified on Boruta algorithm were then used as predictors in a random Forest classification algorithm^13^. The variables which remained significant after this second round of feature selection were analysed separately using robust ANOVA methods and post-hoc tests if the ANOVA was significant. An ANOVA on the medians was also performed for comparison to the robust ANOVA on trimmed means (5% trim).

Adverse events were counted and classified as those occurring during the procedure or in recovery, and those occurring after the procedure, assessed at 72 h and 14 days after the bronchoscopy. Adverse events in different clinical groups were compared using a two-sided Cochran-Armitage test in one-against-the-rest scenarios.

## Results

### Participant characteristics

A total of 337 bronchoscopy procedures were performed solely for research purposes as part of five NIH-funded studies from 1 January 2019 to 15 March 2022 and were included in the analysis. The pertinent characteristics of the study population are described in Table 1.

The most common comorbidity was hypertension (eight in Community Controls, six in Household Contacts, one in the TB groups and four in PLHIV). Four participants had asthma, four had previous chest trauma, and three had allergic rhinitis. The remaining comorbidities were a wide range of minor ailments. Approximately one fifth of the population used recreational substances, of whom 59 (85.5%) smoked cannabis (only five of these did not also smoke tobacco); four (5.8%) used both cannabis and another substance such as methamphetamine or methaqualone (all current tobacco smokers); and six (8.7%) used methamphetamine or methaqualone alone or in combination (all current tobacco smokers).

Of the 78 participants with active TB (the TB Pre-Treatment and TB Early Treatment groups), the mean Body Mass Index (BMI) was 19.5 (14.8–29.0), with 39 (50%) of participants classifying as ‘underweight’ and only four (5.1 %) as ‘overweight’. On study entry, 39 (50.0%) participants were sputum smear positive, with scores as follows: five (6.4%) ‘scanty’ (less than 10 AFB in all fields); 17 (21.8%) ‘1 +’ (less than 1 AFB per field); 13 (16.7%) ‘2+’ (1 to 10 AFB per field); and four (5.1 %) ‘3+’ (more than 10 AFB per field). Of the 76 who had sputum MGIT culture, 71 (93.4%) were culture-positive, with a mean time to positivity of 10.6 days. Of the 70 that had sputum Xpert Ultra results, 66 (84.6%) were positive, with the following ranking: 17 (24.3%) ‘high’; nine (12.9%) ‘medium^1^; 13 (18.6%) ‘low’; six (8.6%) ‘very low’; three (4.3%) ‘trace’; 18 unknown. All participants were symptomatic of TB, but well enough to undergo bronchoscopy. All chest radiographs in the active TB groups were highly suggestive of active TB, except for one participant with a normal chest radiograph. In the TB End of Treatment group, 14 (63.6%) had abnormal radiographs not in keeping with active TB; four (18.2%) had radiographs suggestive of active TB; and only four (18.2%) had normal radiographs. Of the Household Contacts group, 20 (35.1%) had abnormal chest imaging (including both chest radiograph and PET-CT) which was not suggestive of TB, and 11 (19.3%) had chest imaging which was suggestive of TB (either related to previous TB episodes, or implying possible subclinical TB, as participants were asymptomatic and tested negative for TB on sputum). Only nine (6.0%) Community Controls had abnormal imaging (radiographs and PET-CTs), none were suggestive of TB. There were 28 PLHIV included, one of whom had two procedures. Using a composite of both chest imaging modalities, 12 (42.9%) of the PLHIV group were normal, three (10.7%) had results consistent with subclinical TB, 14 (50%) were abnormal but not in keeping with TB. All PLHIV were on antiretroviral therapy (ART) at the time of bronchoscopy, for a median of 10 years duration (range 0.5–17 y). Of the 18 participants who had CD4 counts within six months before the bronchoscopy, the median was 602 cells/μL. Of the 16 participants with HIV viral loads within six months, the median was < 20 copies per millilitre.

### SARS-CoV-2 positive subgroup

The distribution of SARS-CoV-2 participants across clinical groups is shown in Table 1. Of the 42 participants (43 procedures), 31 (72.1 %) were females, and the mean age was 36 years. There were similar proportions of current (20; 46.5%) and never (18;41.9%) smokers. The majority (37; 86.0%) did not use recreational substances, four (9.3%) used cannabis, and two (4.7%) used methamphetamines. Fourteen (32.6%) had a comorbidity, of which the most common was hypertension. Using a composite of both chest imaging modalities, 15 (34.9%) were normal, six (13.9%) were classified as in keeping with active TB, six (13.9%) had subclinical TB, and 16 (37.2%) were abnormal but not in keeping with active TB. Forty participants (95.2%) tested either anti-N antibody positive, anti-S antibody positive, or both. Four (9.5%) tested RT-qPCR positive in the three weeks prior to the bronchoscopy, however none of them had symptoms of coronavirus disease-2019 (Covid19) at the time of testing. Only 16 participants (38.1 %) reported symptoms of Covid19 at any time (mean time between symptoms and bronchoscopy was 6.4 months; in 18 symptom status was unknown); two of these (4.8% of the group) required hospital admission; none of them had active TB.

### BAL yields

**Figure 2:**
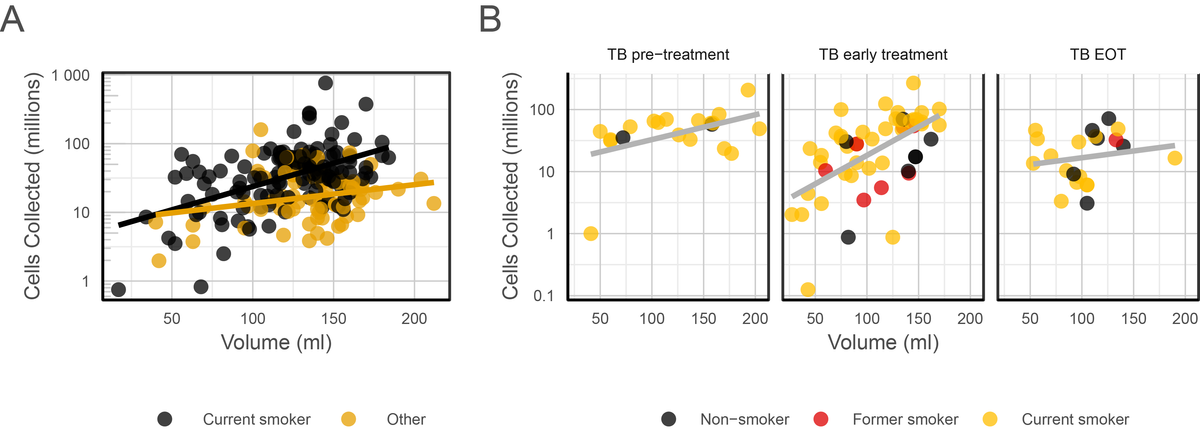
Scatter plot with regression trends showing the relationship between BAL total cell count and BAL volume yield. (A) The correlation between the cell count and BAL volume in the Household Contacts and Community Control groups (the two groups without any current active infection), stratified by smoking status (‘current’ and ‘other (prior smokers and never smokers). The trend line for current smokers indicates an increase of 0.61×106 cells collected per ml increase in volume (SE 0.21×10^6^ cells, P = 0.004) (B) Correlation between cell count and BAL volume among TB groups. The cell yield increases by 0.32×10^6^ cells (SE 0.15×10^6^, P = 0.044) per ml BAL volume among participants before TB Pre-Treatment (TB pre-treatment), by 0.54×10^6^ cells (SE 0.14×10^6^, P < 0.001) per ml among TB Early Treatment participants, and only 0.10×10^6^ cells (SE 0.13×10^6^, P = 0.451) per ml BAL volume among participants at End of Treatment (TB EOT).

Overall, the median BAL volume collected from the procedures was 130 ml (IQR 49 ml), which represents 54.2% of the instilled volume. The median number of BAL cells collected from the procedures was 30.2 × 10^6^ (IQR 38.7 × 10^6^). The median BAL cell concentration (cells/ml) was 0.24 × 10^6^ (IQR 0.34 × 10^6^). The median cell viability (proportion of live cells in the total cell yield) was high, at 96.8% (IQR 2.8%). The analysis showed no significant variability in BAL cell yield over time overall, or when stratified by parent study. There was no significant difference in BAL cell yields between operators performing the bronchoscopy, and no significant variability in their performance over time (Supplementary Fig. S1). We investigated the effect of the BAL volume on the total BAL cell yield and found that when the whole sample was considered there was no significant relationship between the two outcomes. However, when we performed group-specific testing, we found linear relationships in current smokers from the Household Contacts and Community Controls (P = 0.04) and in the TB treatment groups (*p*< 0.0001 for TB Early Treatment and P = 0.044 for TB Pre-Treatment. This suggests that the true relationship was confounded in sample population analysis, as shown in Fig. 2. There were 333 data points available for the pellet colour analysis: 215 (64.6%) pellets were black, 54 (16.2%) were clear, and 65 (19.5%) were bloody.

### Factors affecting BAL yields

**Figure 3:**
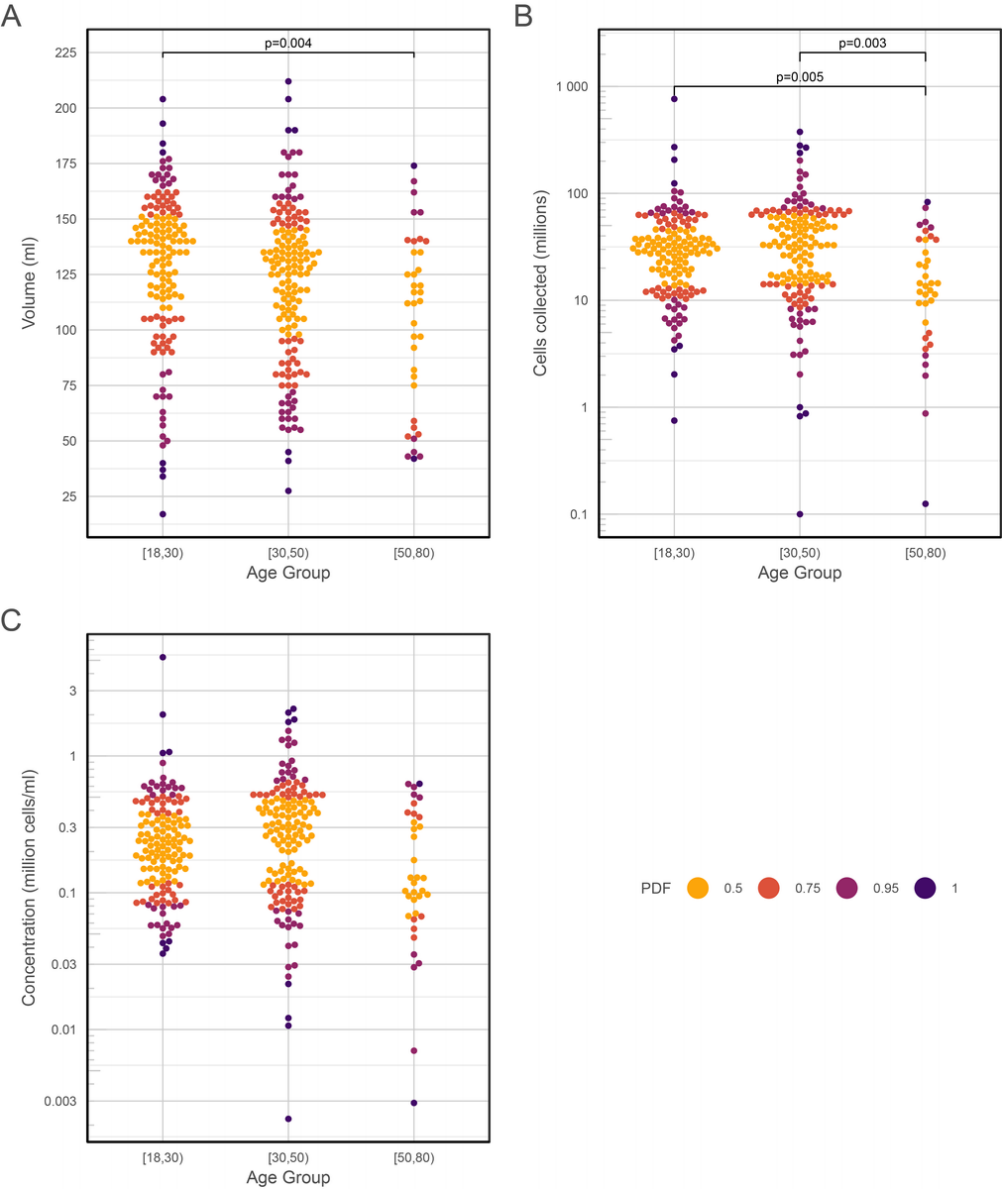
Bee swarm plots showing the effect of participant age on BAL cell yield, volume, and cell concentration. Age was binned into three categories (18 – 30, 30 – 50 and 50 – 80 y). (A) Volume yield; (B) Cell yield; (C) Cell concentration. PDF, a distance metric based on the probability density function showing distance from the mean. ANOVA on medians: (A) F = 4.2957, P = 0.0195; (B) F = 8.202, P = 0.001; (C) F = 5.2743, P = 0.004.

In a simple linear model assessing the impact of the available clinical variables on BAL volume yield, the factor with the greatest effect was the anatomical lung lobe chosen, with the RML yielding significantly higher volumes compared to all other lobes except the lingula (with RML as reference, RUL − 35.4 ml, P< 0.0001; LUL − 22.5 ml, P = 0.001; LUL − 30.8 ml, P = 0.022; RLL − 25.6 ml, P = 0.042; lingula − 12.0 ml, P = 0.198). Increasing age was associated with a lower volume yield (P < 0.0001), as was male gender (P = 0.0447) though the latter became non-significant after correction for multiple testing. When participant age was divided into three categories, pairwise comparisons (ANOVA on the medians) showed a relationship between these categories and BAL volume, cell yield and concentration, which remained significant after correction for multiple testing (Fig. 3).

**Figure 4:**
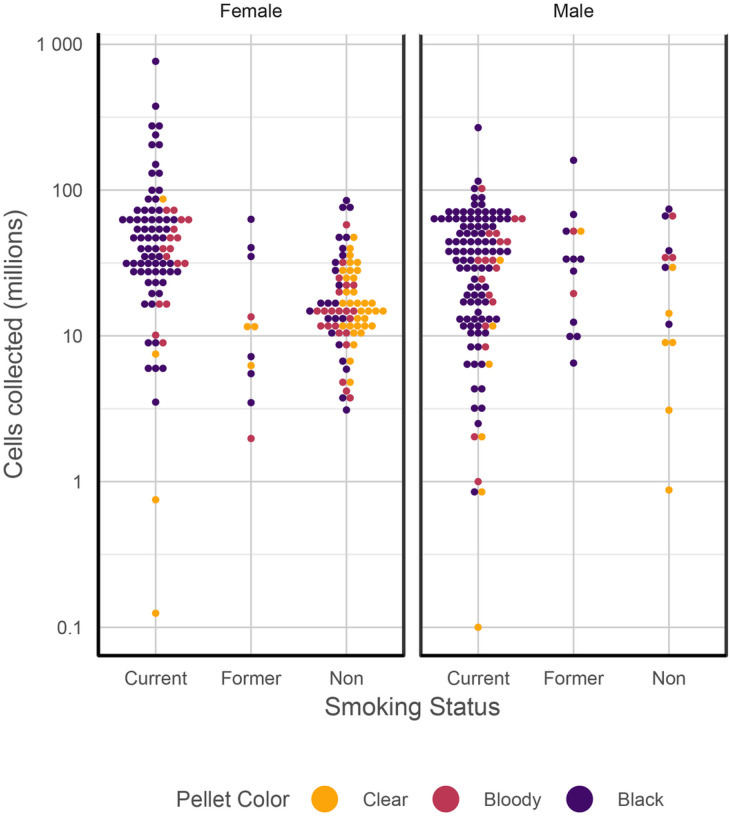
Bee swarm plots showing the relationship between BAL pellet colour, smoking status and gender.

Three variables were highly associated with total BAL cell yields: current smoking (26×10^6^ cells more than never smokers, SE 7.5×10^6^, P < 0.0001); male gender (16×10^6^ cells more than female gender, SE 5.9×10^6^, P = 0.007); and pellet colour (bloody pellet 19.6×10^6^ fewer cells than black pellets, SE 7.3×10^6^, P = 0.0119; clear pellet 20.1×10^6^ fewer cells than black pellets, SE 9.0×10^6^, P = 0.027). The findings for BAL cell concentration were similar. The effect of gender did not stand up to correction for multiple testing, and further interrogation found that the three variables smoking, gender and pellet colour, were highly correlated, explained by the fact that significantly more males were smokers, and smoking is the commonest cause of a black BAL pellet (Fig. 4).

**Figure 5:**
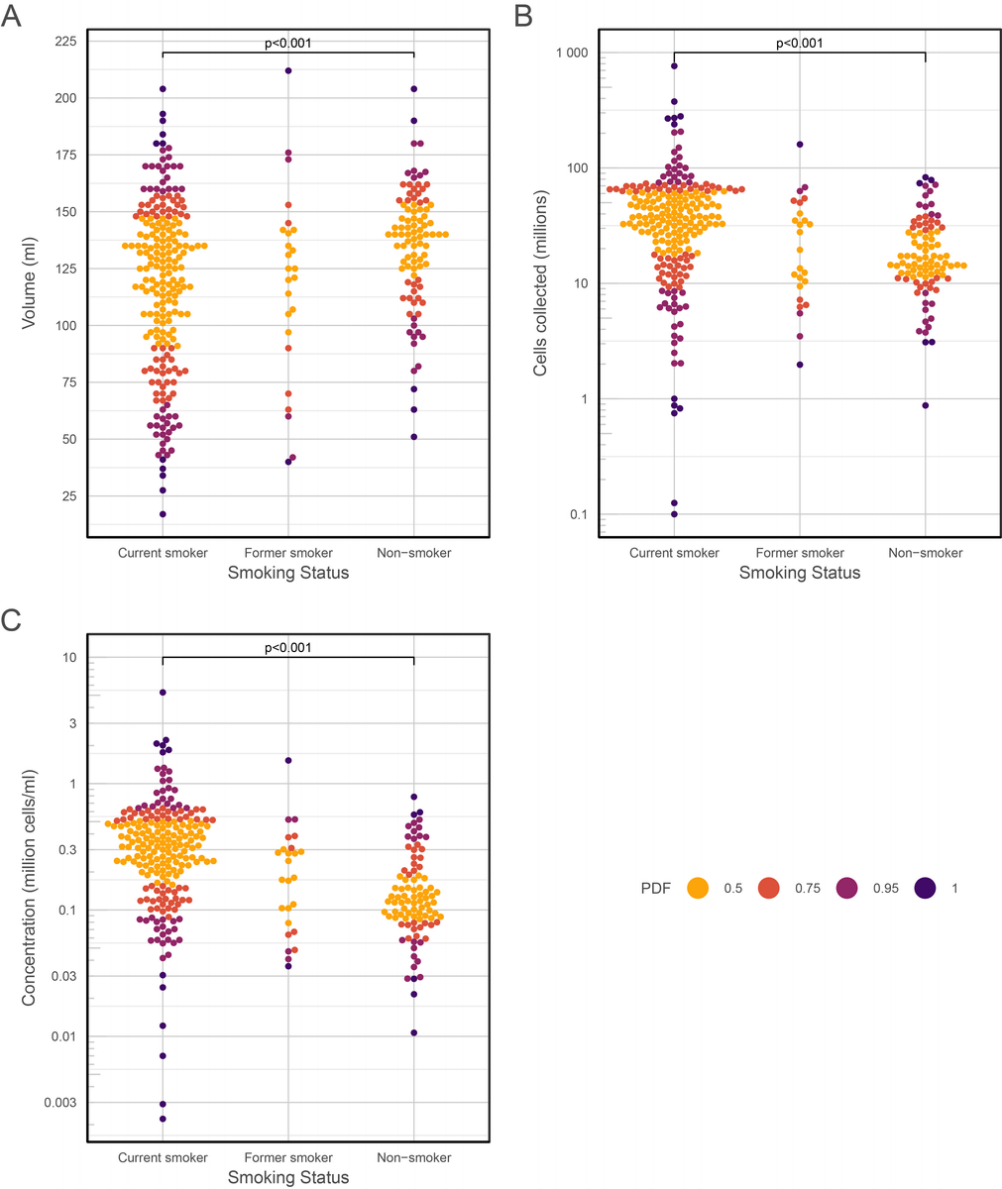
Bee swarm plots showing the effect of smoking on BAL volume, cell yield and cell concentration. (A) Volume yield; (B) Cell yield; (C) BAL cell concentration. PDF, see Fig 3. ANOVA on medians: (A) F = 6.6511, P = 0.0075; (B) F = 25.754, P < 0.001; (C) F = 31.0721, P < 0.001.

To further investigate the effects of smoking on BAL characteristics, pairwise comparisons (ANOVA on the medians) were performed, showing clearly that although current smokers give the lowest BAL volume yields, they have the highest overall cell yields (Fig. 5). These effects were seen for the black BAL pellet category as well.

**Figure 6:**
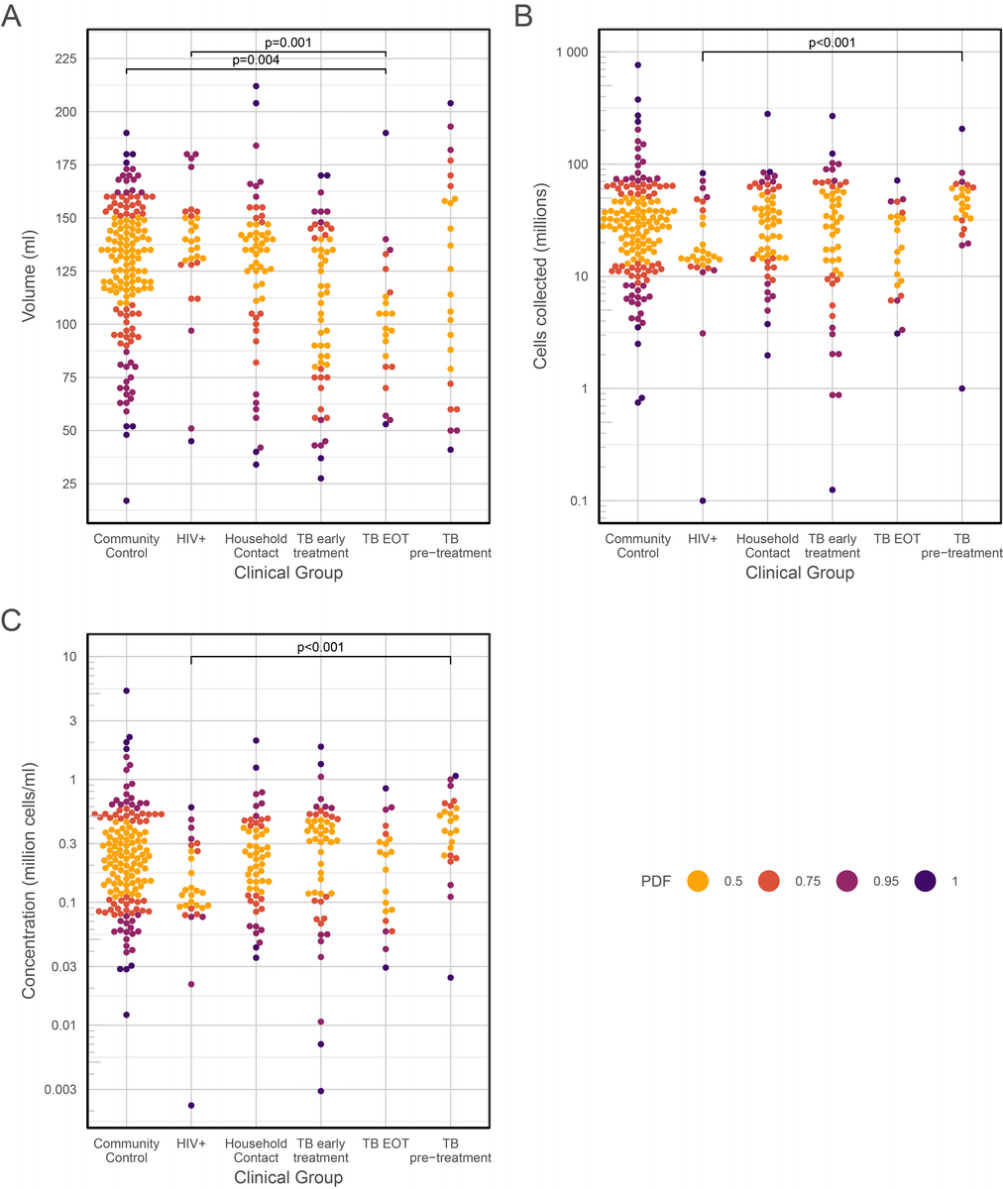
Bee swarm plots showing the effect of clinical group and TB treatment status on BAL volume, cell yield and cell concentration. (A) Volume yield; (B) Cell yield; (C) cell concentration. PDF, see Fig 3. ANOVA on medians: (A) F = 3.8874, P = 0.0015; (B) F = 4.7474, P = 0.001; (C) F = 4.2271, P = 0.0015.

Pairwise comparisons (robust 2-way ANOVA on the medians) revealed some interesting trends in BAL volume and cell yields, from participants* TB treatment status (Fig. 6).

The TB Early Treatment group and the TB End of Treatment group yielded lower BAL volumes than the Community Controls group [using the Community Controls as a reference, TB Early Treatment: −20.3 ml, 95% C.I. (−36.5, −4.06), P = 0.006; TB End of Treatment: −28.4 ml, 95% C.I. (−47.5, −9.28), P = 0.002], but the cell yields and concentrations in the TB Pre-Treatment group were higher than in the Community Controls (total BAL cells: 41.9×10^6^ cells more than in Community Controls, 95%C.I. 18.6–68.8×10^6^, P < 0.0001; BAL cell concentration: 0.44×10^6^ cells/ml more than in Community Controls, 95%C.I. 0.25–0.66×10^6^, P< 0.001). The TB Pre-Treatment group had a significantly higher cell yield (23.7×10^6^ cells more than the TB End of Treatment group, 95%C.I. 7.45–40×10^6^, P = 0.002) and concentration (0.2×10^6^ cells/ml more than the TB End of Treatment group, 95%C.I. 0.01–0.4×10^6^, P = 0.035) than the End of Treatment group, suggesting the TB treatment duration affected the cellular content of the BAL. The trend was confirmed on direct comparison of paired Early Treatment and End of Treatment samples (P = 0.025), and remained significant when the results were adjusted for smoking status. The apparent effect of HIV on lower BAL cell yields seen in Fig. 6 was confounded by most of these participants being never-smokers.

Variables which had no effect on the outcomes in the linear models included SARS-CoV-2 status, imaging findings (using a composite of chest radiograph and PET-CT when performed, categorised as ‘normal’, ‘abnormal, in keeping with active TB’, or ‘abnormal but not active TB’), any airway abnormalities observed during the procedure, IGRA result, recreational substance use, and the presence of other comorbidities. Interestingly, ANOVA on the medians revealed the following interactions: participants who had airways which collapsed on minimal suction during the procedure (suggesting underlying airways disease) had a lower volume yield than those with no observable airways abnormalities (P < 0.001); the presence of abnormal secretions (suggesting chronic lung disease or current infection) also predisposed to lower volume yield (P = 0.007); and both abnormal imaging categories (‘abnormal but not active TB’, and ‘abnormal, in keeping with active TB’) were associated with a lower BAL volume yield than those with normal imaging results (P = 0.004).

### Factors affecting BAL pellet colour

**Figure 7:**
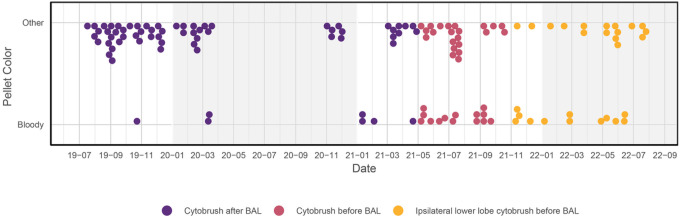
The effect of bronchial brushings on BAL pellet colour. This time series shows the effect of performing a same segment bronchial brushing with a ‘cytobrush’ after the BAL compared to performing it in the same segment or in the ipsilateral lower lobe before the BAL. Dates in ‘YY-mm’ format (last two digits of year and a two-digit month). The Cochran-Armitage test for trends gave P < 0.001.

ANOVA testing showed that the highest cell counts are found in black pellets, followed by bloody pellets, then clear pellets (P< 0.001). While red blood cells may obscure black particulate in pellets that would otherwise be labelled black (and thus explain the higher cell counts in bloody pellets), if the bloody pellet’s higher cell count represents contamination with blood immune cells, this has potentially negative implications for the post-thaw BAL cell viability and the interpretation of any BAL immunoassays. Logistic regression analysis found that being in the PLHIV group was associated with a bloody pellet (P = 0.013). As the only two parent studies to include PLHIV also had brushings during their procedures, we investigated whether the order in which the procedures were performed had any effect on the pellet colour and found that performing the brushing after the BAL (in the same lung lobe) was associated with a significantly lower rate of bloody BAL pellets compared to performing the brushing before the BAL (P = 0.001). As the reduced visibility and enhanced cough stimulus after the BAL may compromise participant safety when performing a brushing, our group opted to try a third technique in sampling the ipsilateral lower lobe before the BAL, but this was not successful in reducing the proportion of bloody pellets (Fig. 7).

### BAL cell differential count

It has been shown that smaller instilled BAL volumes sample only the distal airways whereas larger volumes sample the alveolar compartment, resulting is differences in measured protein and cellular contents of BAL fluid.^3^ We therefore investigated whether the BAL volume yield affected cell differential counts, but found that there was no relationship between the two. On pairwise ANOVA on the medians, several significant differences in the differential cell counts between groups were found: 1) older age categories were associated with higher neutrophil proportions (P = 0.003); 2) the TB Early Treatment group had the highest proportions of both neutrophils and eosinophils of the clinical groups (P = 0.03 and P = 0.045, respectively); 3) clear BAL pellets had the highest proportions of lymphocytes (P = 0.002), black pellets had the highest proportions of neutrophils (P = 0.01), and bloody pellets did not have any significantly enriched cell populations; 4) never smokers had the highest proportions of lymphocytes (P = 0.045); ANOVA on means found that current smokers had significantly higher proportions of neutrophils and eosinophils, but this interaction was not significant on comparison of medians; and 5) SARS-CoV-2 positive participants had higher proportions of BAL lymphocytes than SARS-CoV-2 negative participants (P = 0.023). The median BAL differential cell counts in each clinical group are presented in Supplementary Table S2.

### Adverse events

The mean procedure duration (counted from intubation with the bronchoscope to extubation) was 11.5 minutes (range 4–22), with a mode of 10 minutes. Procedures in which a brushing was done with the BAL were significantly longer than those without (P < 0.01); the two studies including brushings had more procedures lasting >12 minutes (P < 0.05), > 14 minutes (P < 0.05) and >15 minutes (P < 0.01).

There were no severe adverse events in this study. Minor adverse events were recorded for 118 (33.1 %) participants. Supplementary Figure S2 shows the minor adverse events, stratified by clinical group. Minor bronchial mucosal bleeding was significantly more common in the PLHIV group (P< 0.001), all of whom also had brushings in the same procedure. Overall, 69 (23.5%) of 293 participants with available data reported still experiencing symptoms at 72 h after bronchoscopy (Supplementary Figure S3). ‘Throat discomfort^1^ and post procedure cough were significantly different between groups, being less frequently reported in the TB Pre-Treatment group (P = 0.0264), TB Early- and End of Treatment groups (P = 0.044) respectively. PLHIV were more likely to experience dizziness than other groups (P = 0.010).

Of those who experienced any symptom, eight (2.7%) sought medical attention for the problem. One (0.3%) study participant was admitted overnight to hospital, three (1.0%) received antibiotics, and six (2.0%) received other medications (including anti-inflammatories, antihistamines, steroids or analgesics). At the day 14 assessment, no participants reported any additional problems. Importantly, at the 72 h assessment, 234 (96.7%) of the 242 participants surveyed stated they would be willing to have a repeat procedure; 236 (97.5%) reported that their overall experience of the bronchoscopy was tolerable; and 239 (98.8%) participants reported that the bronchoscopy procedure was better than they expected. Results were similar at the day 14 assessment.

## Discussion

Our findings show that in a sample of over 330 research BAL procedures across diverse clinical groups made up of predominantly current tobacco smokers, the research team may expect a median yield of approximately 130 ml BAL fluid, or 54% of instilled volume, containing 30 million immune cells, with some exceptions. Using our methods, the pre-cryopreservation cell viability was consistently high between procedures, laboratory personnel and bronchoscopy operators. The procedures conducted in a fully equipped, dedicated bronchoscopy suite were safe, with no severe adverse events, with only 2.7% of participants experiencing symptoms which required medical attention after the bronchoscopy. Performing brushings before the BAL, in the same segment or in the ipsilateral lower lobe, is not advised because of the high rate of blood contamination compared to performing it after the BAL, when the risk of serious adverse events is theoretically higher. Our current practice – informed by these results – is to do the brushing after the BAL, with predetermined safety checks both during the procedure and afterwards, including both a thoracic ultrasound and prolonged observation period.

Consistent with the existing guidelines, the RML yielded the highest BAL volume. However, the factors determining how many cells were present in that volume of fluid were more complex. Smoking appeared to be the single most important factor affecting yields from these procedures. Although current smokers gave consistently lower volume yields than never smokers, their immune cell yields were significantly higher — presumably across all cell types except lymphocytes (which were highest in never smokers) — and increased proportionately with volume yield. Participant age appears to be another important factor. Increasing age was associated with not only lower volume yield but also lower cell yield (as well as lower cell concentration, indicating that the drop in cells is independent of the drop in volume), and a higher proportion of neutrophils within their cell population. Our findings confirm those of Koda and colleagues^14^ who identified an anatomical lung lobe target other than the RML or lingula, having a high pack year smoking history and older age as being negatively correlated with BAL volume yield, which they defined as a less than 30% return of instilled BAL volume in their group of 401 patients with diffuse parenchymal lung disease. Olsen *et a*.^15^ found that there was a similar age-dependent decrease in the BAL volume yield, but, in their group of 295 never-smoking healthy volunteers, the cell yield and differential counts were independent of age, gender, season and anatomical lung lobe. Karimi *et al*.^7^ compared the cell yields in 132 current smokers, 44 prior-smokers and 295 never smokers and concluded that BAL cell recovery was dependent on cumulative smoking history, which they also quantified in pack years. Whilst the pack-years information was available to us in this analysis, in our experience this metric is highly unreliable as an objective measure of smoking intensity, and we therefore chose to exclude it. Karimi *et al*.^7^ also found that BAL volume recovery was negatively correlated with age, and interestingly that smoking men had a lower BAL volume recovery than smoking women^7^. Men also yielded lower volumes and cells in our results, though neither of these stood up to correction for multiple testing and the high correlation between gender and smoking status suggested this finding was more likely attributable to the difference in smoking status between genders.

The effects of TB on these outcomes were interesting: participants with TB who were untreated or early in treatment yielded the lowest volumes but highest cell counts, with higher proportions of granulocytes than the other clinical groups, and a steep proportional increase in the number of cells with increasing volume. The practical outcome is that a participant with TB may disappoint the bronchoscopist with a mere 80 ml volume yield, but within that volume are more immune cells than a ‘decent’ 130 ml yield from a healthy Community Control participant, particularly if that individual is a non-smoker. This was not the case for participants who had reached the end of their TB treatment. In general, those at the end of treatment had both lower volume and lower cell yields, possibly attributable to chronic TB-related airway inflammation and damage reducing the volume return, but with little residual active inflammation at the time of lavage, and so fewer immune cells. A previous publication from our group which examined the performance and immune characteristics of BAL in 58 participants with active TB, other lung diseases, household contacts and healthy controls, found a similar significantly lower BAL volume and higher BAL cell yield in the TB group compared to the others, with higher proportion of neutrophils^16^, but the current study is the first to provide information on the effects of treatment on these outcomes. An important caveat is that the observed differences in cell differential counts may not translate into differences in immune cell phenotypes and function.

From the data one might infer that HIV infection results in a high volume yield with low immune cell content, which has a high proportion of lymphocytes, but there may be a selection bias to consider, as the group of PLHIV that was included were mainly non-smoking middle-aged women who were well-controlled on ART. The lung is a known reservoir for HIV, resulting in pulmonary immune dysregulation, chronic inflammation, and a higher rate of infectious and non-infectious lung diseases^17^. Since the dysregulation is only partially restored by ART, our findings would need to be confirmed by a comparison to participants with untreated HIV, smokers living with HIV, and never-smoking community controls. Performing BAL on participants with untreated HIV may, however, result in a higher rate of post-procedural infections, representing a real safety risk for these people, and it should therefore probably be avoided outside of a clinical context^18^.

Reassuringly for research projects in the post- Covid-19 pandemic era, there were no significant observable effects of recent mild SARS-CoV-2 infection on our BAL yield outcomes, other than a slightly higher proportion of lymphocytes in the BAL fluid. Importantly though, we cannot make the same conclusion across all severities of SARS-CoV-2 infection, and since no functional assays were included in this analysis it remains unclear how the host lung immune profile is affected by recent SARS-CoV-2.

Overall, our study was limited by the need to select generally healthy candidates for bronchoscopy, potentially excluding important groups such as those with advanced HIV infection and the elderly. As demonstrated above, our results are confounded by numerous important and interrelated variables including the participant’s smoking status, recreational drug use, whether a brushing was done in the same procedure, and even age and gender in some groups. Teasing apart the individual effects of these variables on the BAL outcomes is complex, and there may be additional confounders which we have not accounted for. Lastly, our BAL cell viability was only assessed pre-cryopreservation, and post-thaw viability is yet to be established for these samples.

## Conclusions

Bronchoscopy with BAL is an invaluable research tool in the study of host immune responses to respiratory infectious disease. Our results show that BAL for research purposes alone, without anticipated clinical benefit, is safe, and even in the presence of current or recent infection with TB, HIV and SARS-CoV-2, the BAL volume yield, cell yield, concentration and viability are consistent and reasonably predictable. Researchers specifically need to consider their target population’s smoking status (and possibly gender), age range, HIV status, and duration on TB treatment when selecting end assays which require a large number of immune cells. Clinicians performing the bronchoscopy should wherever possible aim for the RML (with lingula as an acceptable alternative), anticipate a lower volume yield and consider a higher instilled BAL volume wherever a different lobe is targeted. Additionally, if a brushing is planned during the same procedure, then a strategy to avoid contaminating the BAL with blood whilst optimising participant safety, should be identified beforehand. Whether the clinical variables which this study has identified as important in determining BAL volume yield, BAL cell yield, and pre-cryopreservation viability also impact the immune cell phenotype, function, and post-thaw viability, is yet to be established.

## Figures and Tables

**Table 1:** Characteristics of the study population stratified by clinical group.

	Community Controls (n = 150)	Household Contacts (n = 57)	TB Pre-Treatment (n = 24)	TB Early Treatment (n = 54)	TB End of Treatment (n = 22)	PLHIV (n = 29)	Pooled (n = 337)
Age in years	29 (16.8)	34 (17.8)	32 (15.3)	33 (21.5)	31 (17.3)	44 (13.5)*	32 (20)
Female	89 (59.3)	36 (66.7)	7 (35.0)	14 (25.9)	6 (27.3)	25 (86.0)*	178 (54.1)
Current tobacco smoking	101 (67.3)	31 (57.4)	18 (90.0)	40 (74.0)	15 (68.2)	7 (24.1)*	212 (64.4)
Recreational substance use	21 (14.0)	14 (25.9)	6 (30.0)	19 (35.2)	7 (31.8)	2 (6.9)	70 (21.3)
SARS-CoV-2 positive^+^	17 (11.3)	14 (24.6)	6 (25.0)	0	0	6 (20.7)	43 (15.9)
Other comorbidity^$^	24 (16)	9 (16.7)	1 (5.0)	8 (14.8)	1 (4.5)	4 (13.8)	47 (13.9)
IGRA status^#^ positive negative	101 (67.3) 41 (27.3)	48 (88.9) 6 (11.1)	ND	ND	ND	21 (72.4) 8 (27.6)	184 (55.9) 57 (17.3)

Age is expressed as median (interquartile range); all other variables are expressed as number followed by the percentage (in parentheses) that this number represents of valid samples in the specific study group for the specific variable.

* Two-sided Cochran-Armitage tests found no statistically significant differences between the first five clinical groups. On ANOVA, age was significantly different between PLHIV and the other clinical groups (P < 0.001, F-statistic = 7.842). Fisher’s exact test found that the PLHIV group had significantly more females (P < 0.001, 99.5% C.I. for odds ratio 1.77–107.9) and fewer current smokers (P < 0.001, 99.5% C.I. for odds ratio 0.03–0.5) than the other clinical groups.

+ One study only performed SARS-CoV-2 RTPCR, not serology, and thus participants were unable to be classified. The unclassified participants were not included in the analysis for this group.

$ Excluding TB, HIV, and previous SARS-CoV-2 infection.

# Several participants’ IGRA results were ‘indeterminate’ or unknown; IGRA was not performed for participants with active TB.

IGRA, interferon gamma release assay; ND, not determined; PLHIV, people living with HIV; SARS-CoV-2, severe acute respiratory syndrome coronavirus 2;TB, tuberculosis.

## Data Availability

The datasets generated and analysed during the current study are available from the corresponding author on reasonable request.
